# Influences of diet and the gut microbiome on epigenetic modulation in cancer and other diseases

**DOI:** 10.1186/s13148-015-0144-7

**Published:** 2015-10-16

**Authors:** Bidisha Paul, Stephen Barnes, Wendy Demark-Wahnefried, Casey Morrow, Carolina Salvador, Christine Skibola, Trygve O. Tollefsbol

**Affiliations:** Department of Biology, University of Alabama at Birmingham, 175 Campbell Hall, 1300 University Boulevard, Birmingham, AL 35294-1170 USA; Department of Pharmacology and Toxicology, University of Alabama at Birmingham, Birmingham, AL USA; Department of Nutrition Sciences, University of Alabama at Birmingham, Birmingham, AL USA; Department of Cell, Developmental and Integrative Biology, University of Alabama at Birmingham, Birmingham, AL USA; Division of Medical Oncology/Hematology, University of Alabama at Birmingham, Birmingham, AL USA; Comprehensive Cancer Center, University of Alabama at Birmingham, Birmingham, AL USA; Comprehensive Center for Healthy Aging, University of Alabama at Birmingham, Birmingham, AL USA; Nutrition Obesity Research Center, University of Alabama at Birmingham, Birmingham, AL USA; Comprehensive Diabetes Center, University of Alabama at Birmingham, Birmingham, AL USA; Department of Epidemiology, University of Alabama at Birmingham, Birmingham, AL USA

**Keywords:** Epigenetics, Epigenome, Methylation, Acetylation, Histone proteins, Gut microbiome

## Abstract

Epigenetic modulation of gene activity occurs in response to non-genetic factors such as body weight status, physical activity, dietary factors, and environmental toxins. In addition, each of these factors is thought to affect and be affected by the gut microbiome. A primary mechanism that links these various factors together in mediating control of gene expression is the production of metabolites that serve as critical cofactors and allosteric regulators of epigenetic processes. Here, we review the involvement of the gut microbiota and its interactions with dietary factors, many of which have known cellular bioactivity, focusing on particular epigenetic processes affected and the influence they have on human health and disease, particularly cancer and response to treatment. Advances in DNA sequencing have expanded the capacity for studying the microbiome. Combining this with rapidly improving techniques to measure the metabolome provides opportunities to understand complex relationships that may underlie the development and progression of cancer as well as treatment-related sequelae. Given broad reaching and fundamental biology, both at the cellular and organismal levels, we propose that interactive research programs, which utilize a wide range of mutually informative experimental model systems—each one optimally suited for answering particular questions—provide the best path forward for breaking ground on new knowledge and ultimately understanding the epigenetic significance of the gut microbiome and its response to dietary factors in cancer prevention and therapy.

## Background

There is intense ongoing research activity to elucidate the relationship between the human microbiome and various diseases including cancer. “Microbiome” is a collective term used for the genes from colonizing organisms including fungi, viruses, and bacteria, the latter of which are by far the most populous. The microbiome are ubiquitous and distinct populations reside in the oral cavity, stomach, upper and lower intestine, urinary tract, genitalia, etc. It is increasingly believed to play a dynamic role in the health of individuals. The gut is inhabited by the largest variety of bacteria, which contribute to the metabolism of different classes of food materials. An overwhelming 100 trillion commensal bacteria live within the human gastrointestinal tract, constantly exposing the surface of the intestinal mucosa to the stimulatory effects of this resident microbiota [86]. Humans are thought to have evolved a symbiotic relationship with the microbiota of the gut; however, the molecular mechanisms remain poorly understood. DNA-based analysis has significantly facilitated the identification of bacteria comprising the microbiome, but understanding function remains more challenging. For example, indigenous microbiota produce low molecular weight (LMW) substances that potentially interact with the tissue cellular environment to modulate signaling pathways and regulate gene expression. Recently, these LMW substances have been shown to influence epigenetic modifications, chromatin remodeling, and other signaling molecules, ultimately regulating apoptosis, cellular differentiation, and inflammation. Therefore, the epigenetic changes brought about by microbiota may contribute to prevention or therapeutic intervention of cancer or other diseases.

### Epigenetics

The molecular mechanism of epigenetics involves many processes such as DNA methylation, posttranslational modification of histone proteins, silencing of the extra copy of the X-chromosome in females, and genomic imprinting. Non-coding RNAs including microRNA, small interfering RNA, and long non-coding RNA are also players that fall under the broader definition of epigenetics [51].

#### DNA methylation

**Fig. 1:**
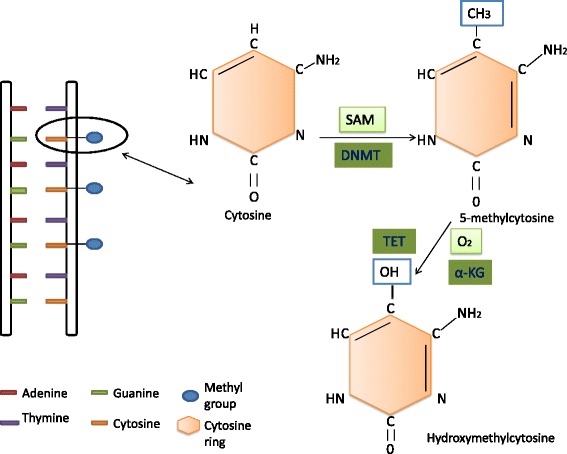
The basic process of DNA methylation. *S*-adenosylmethionine (*SAM*) is used as the methyl (*CH3*) donor by the enzyme DNA methyltransferase (*DNMT*) to transfer a methyl group to cytosine rings of the DNA strands. TET proteins are dioxygenase enzymes that hydroxylate 5-methylcytosine residues to form 5-hydroxymethylcytosine (5hmC). They use a metabolite intermediate, α-ketoglutarate (*α-KG*), and molecular oxygen as enzyme cofactors for this reaction

DNA methylation is the addition of a methyl group to the carbon-5 position of the cytosine pyrimidine ring and occurs predominantly in cytosine guanine dinucleotide-rich regions known as CpG islands that are found in the 5′ regulatory regions of most genes. The enzymes involved in DNA methylation are called DNA methyltransferases (DNMTs). TET (10-11 translocation) proteins are dioxygenase enzymes that hydroxylate 5-methylcytosine residues to form 5-hydroxymethylcytosine (5hmC). They use a metabolite intermediate, α-ketoglutarate (α-KG), and molecular oxygen as enzyme cofactors for this reaction [65]. Bacteria can cause changes in DNA methylation patterns of host cells by providing epigenetically active metabolites such as folate, butyrate, and acetate. These metabolites are essential for DNA methylation. For example, folate (produced by *Bifidobacterium* spp.) is a methyl donor and is necessary for generation of *S*-adenosylmethionine (SAM) which in turn is a methyl-donating substrate for DNA methyltransferases [28]. The presence of 5hmC at bivalent loci and DNaseI hypersensitive sites (HS) might imply that simultaneous removal as well as renewed deposition of 5mC by DNMTs is taking place [55]. Thus, DNA methylation is a dynamic event and several cancers have been associated with this positional alteration of DNA methylation [29]. During carcinogenesis, epigenetic switching and 5-methylcytosine reprogramming result in the aberrant hypermethylation of CpG islands, leading to the unresponsiveness of tumor suppressor genes [85]. In cancers with a stem cell origin, isocitrate dehydrogenase (IDH) mutations result in the production of oncometabolite 2-hydroxyglutarate (2HG), leading to global hypermethylation, whereas in tumors with non-stem cell origin an intact metabolic function of IDH would be necessary to maintain DNA methylation plasticity [58] (Fig. 1).

#### Chromatin remodeling and histone modification

Histone acetylation is catalyzed by lysine acetyltransferases (KATs), which transfer an acetyl group from acetyl coenzyme A (acetyl-CoA) to lysine residues (Nɛ), with the concomitant production of CoA [73]. Acetylation is primarily associated with transcriptional activation, and most of the acetylation sites occur within the N-terminal tail of the histones. Acetylation of the histones leads to increased accessibility of nucleosomal DNA to transcription factors. Histone deacetylases (HDACs) are opposite in function to histone acetyltransferases (HATs) as they remove the acetyl (acyl) moiety from lysine residues. Histone acetylation is regulated via the tricarboxylic acid (TCA) cycle. The gut microbiome produces short-chain fatty acids that are used to generate ATP via the TCA cycle. HDACs can also be inhibited by metabolites produced by the gut microbiome such as butyrate and propionate. ATP citrate lyase (ACL) uses mitochondrial-derived citrate to generate acetyl-CoA in the cytoplasm and nucleus. In the nucleus, HATs transfer the acetyl group from acetyl-CoA onto histone lysine [77]. Histone methylation is also an important epigenetic process in that methylation of histones is effective in recruiting certain transcription factors to the chromatin. Lysine and arginine residues can be methylated histone methyltransferases. There are additional histone tail modifications such as histone phosphorylation, ubiquitination, sumoylation, histone poly-ADP ribosylation, histone biotinylation, citrullination, and proline isomerization. Moreover, there is cross-talk not only among the various histone modifications but also between DNA methylation and histone modification that often collectively work in tandem to control gene activity [40]. As for DNA, methylated histones undergo demethylation by the JmjC family of dioxygenases that once again depend on molecular oxygen and α-KG.

#### Non-coding RNA

RNA that does not code for a protein is termed non-coding RNA (ncRNA). It is important to note that, unlike eukaryotes, ncRNAs form a small part of the prokaryotic genome although small ncRNA has been reported in bacteria. By contrast, the majority of the eukaryotic genome is transcribed into ncRNA which plays a major role in the regulation of messenger RNA (mRNA) translation. ncRNA can be divided into small and large ncRNAs. Small ncRNAs, also known as microRNAs (miRNAs), are approximately 18–20 nucleotides in length and play a critical role in gene regulation [38]. Small interfering RNAs (siRNAs) or PIWI-interacting RNAs (piRNAs) are key small ncRNAs that play important roles in several biological processes, especially cancer [18]. Long ncRNAs are 200 nucleotides in length or longer. The broad functional repertoire of long ncRNAs includes roles in high-order chromosomal dynamics, telomere biology, and subcellular structural organization [59]. Exosomes are nanovesicles (30–90 nm in diameter) secreted by intestinal epithelial cells (IECs) either from their apical or basolateral side [83]. Through the exosomes, proteins, lipids, mRNAs, and miRNAs are released. Exosomes carry molecules involved in adhesion and antigen presentation, comprising major histocompatibility complex (MHC) class I and class II molecules, tetraspan proteins, CD26/dipeptidyl-peptidase IV, and A33 antigen, a molecule essentially restricted to the intestinal epithelium [31, 52]. Apical secretion of exosomes into the lumen have an effect on the function of distant cells, whereas basolaterally released exosomes regulate local innate responses [24].

### Epigenetic influence through metabolite production by the gut microbiome

The microbiota of the gastrointestinal tract harbors a huge collection of beneficial symbionts that aid in digestion, as well as provide a source of various nutrients. In most mammals, the gut is dominated by four bacterial phyla that perform these tasks: *Firmicutes*, *Bacteroidetes*, *Actinobacteria*, and *Proteobacteria* [45]. These microorganisms produce a number of LMW bioactive substances such as folate, butyrate, biotin, and acetate that may participate in epigenetic processes [36].

Folate is a vitamin that accepts one-carbon units from donor molecules and is involved in many metabolic pathways, such as methyl group biogenesis and synthesis of nucleotides, vitamins, and some amino acids [69]. The efficiency of DNA replication, repair, and methylation are affected by folate availability; rapidly proliferating cells such as leukocytes, erythrocytes, and enterocytes require large amounts of folate. Folate is widely distributed in the biological world, intestinal bacteria being one source of this vitamin [30].

Butyrate is a short-chain fatty acid (SCFA) and a potent inhibitor of HDACs [12]. The most important butyrate producer is *Faecalibacterium prausnitzii* which belongs to the cluster of *Firmicute* bacteria, comprised of *Clostridium leptum*, *Eubacterium rectale*, and *Clostridium coccoides* [49]. Butyrate has the ability to activate epigenetically silenced genes in cancer cells such as *p21* and *BAK* [6]. Butyrate has been shown to repress angiogenesis in vitro and in vivo and reduces the expression of pro-angiogenic factors such as EGF and HIF 1α [99]. Increasing the concentration of butyrate in the colon can play a protective role and prevent cancer, and its production is dependent on diet and intestinal microflora composition. Butyrate is able to modulate intestinal microflora through regulation of pH and exerts many beneficial effects on the intestinal lumen through epigenetic mechanisms [94]. The HDAC inhibitor sodium butyrate is known to increase cell death in human medulloblastoma cells [62]. We have shown that epigallocatechin (EGCG) obtained from green tea and sodium butyrate given at physiological doses achievable in the human diet induced apoptosis and cell cycle arrest. They were found to be regulated by decreases in the epigenetic-modifying enzymes HDAC1 and DNMT1 as well as survivin in colon cancer cells [75].

Biotin is a vitamin that mammalian cells cannot produce, and they depend on a constant supply of biotin from the intestinal microbiota to maintain normal levels of protein biotinylation. Biotinylation is an important epigenetic process that involves the attachment of biotin to histone proteins resulting in gene repression, and it also plays a role in DNA repair and chromatin structure [79].

At the chromatin level, it has been widely demonstrated that the balance between acetylation and deacetylation of histone and non-histone proteins plays a pivotal role in the regulation of gene expression [67]. HATs and HDACs transfer an acetyl group from acetyl-CoA to the Ɛ-amino group of lysine and remove the acetyl group, respectively. CoA is a by-product in this reaction. The main donor of acetyl groups for formation of acetyl-CoA in acetylation reactions is the gut microbiota [78]. Similar to yeast, acetylation is regulated by metabolic intermediates of glucose, and one major enzyme involved is ATP citrate lyase, which converts citrate produced by the mitochondria into acetyl-CoA [19]. The ratio of acetyl-CoA to CoA is important for the regulation of acetylation in response to metabolic changes [1]. Under conditions of glucose deprivation, the ratio of acetyl-CoA to CoA drops, and this in turn affects histone acetylation levels. Studies in yeast have elucidated that high acetyl-CoA stimulates promoter histone acetylation [5]. This may have important implications in cancer studies as oncogenes may make use of acetyl-CoA metabolism to alter chromatin for growth [8].

**Fig. 2:**
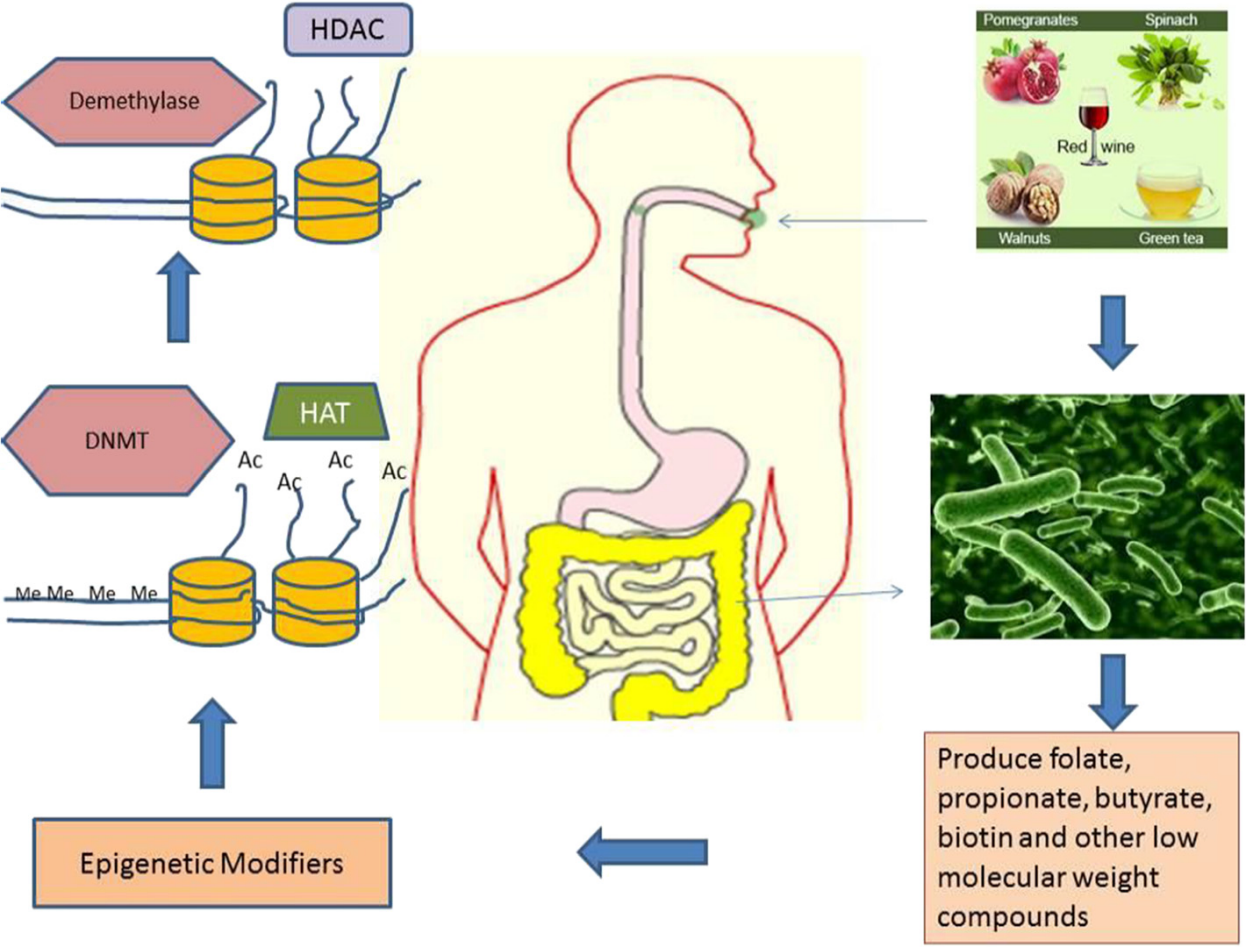
The molecular interaction of the gut microbiota is greatly influenced by the dietary compounds consumed. The microbes residing in the human gut produce a number of low molecular weight molecules such as butyrate, folate, propionate, and biotin. These compounds either directly bring about epigenetic modifications such as changes in DNA methylation and histone acetylation or indirectly act via activation or inhibition of certain enzymes such as DNMTs, HDACs, or even hTERTs. *Me* DNA methylation, *Ac* histone acetylation

The gut microbiota also contribute to the absorption and excretion of minerals such as zinc, iodine, selenium, cobalt, and others that are cofactors of enzymes participating in epigenetic processes. Moreover, various enzymes such as the methyltransferases, acetyltransferases, deacetylases, Bir A ligase, phosphotransferases, kinases, and synthetases are derived from the gut microbiota. A number of key energy metabolites including *S*-adenosylmethionine (SAM), acetyl-CoA, NAD^+^, α-KG, and ATP serve as essential cofactors for many, perhaps most, epigenetic enzymes that regulate DNA methylation, posttranslational histone modifications, and nucleosome position. Significant contributors in the epigenomic machinery are formed during energy metabolism in eukaryotic cell mitochondria and in the prokaryotic cell membrane signifying that any disorder in these processes can lead to a wide variety of diseases associated with epigenetic modifications [80] (Fig. 2).

### Food, the gut microbiota, and epigenetics

It has been shown that maternal and neonatal nutrition have a major impact on the epigenome of the offspring in that the food consumed modulates the composition of the gut microbiota and also the metabolites that they produce [89]. The gastrointestinal tract of the fetus is virtually sterile; colonization begins at birth from the maternal microbiota of the genital tract, colon, and the overall environment [20]. The bacteria identified include members of the genera *Bifidobacterium*, *Ruminococcus*, *Enterococcus*, *Clostridium*, and *Enterobacter*. The composition of the gut microbiota is profoundly influenced by the diet of the infant [66]. In breast-fed infants, the microbiota predominantly consists of *Bifidobacteria*, and a plethora of diverse microbiota develops after the introduction of solid food commences. In contrast, the intestines of formula-fed infants are colonized by members of a variety of bacterial genera, including those of the enterobacterial genera *Streptococcus*, *Bacteroides*, and *Clostridium*, as well as members of the genus *Bifidobacterium* [20]. By the age of 2 years, the bacterial population of the gut is established and remains relatively constant throughout life, though disease processes, surgical interventions, medical therapies, and epigenetic-modulating factors such as the diet can influence the gut microbiome. Recent studies have revealed an association between bacterial predominance and epigenetic profile. A study on pregnant women with *Firmicutes* and *Bacteroidetes* as the dominant groups in their gut revealed a correlation between differential methylation status of gene promoters associated with obesity and cardiovascular disease [42].

The genetic content of an individual’s gut microbial community is dynamic. It has also been observed that the incidence of colon cancer is lower in Asian countries where the diet consists of fruits and vegetables [20]. Microbiota within the colon convert dietary fiber into SCFAs by the process of fermentation, and as discussed previously, one of the predominant SCFAs is butyrate. Butyrate is naturally produced in the colon through microbial fermentation. It can induce cell differentiation [2], apoptosis [25], and histone hyperacetylation [7]; thus, it has potential to be a major chemotherapeutic agent.

High dietary consumption of fat and red meat (especially processed meat) is associated with increased risk of colorectal cancer. This effect is thought to be modulated by *N*-nitroso compounds and heterocyclic aromatic amines [33]. Consumption of red meat delivers L-carnitine to gut bacteria. These bacteria digest L-carnitine and convert it into trimethylamine-*N*-oxide (TMAO). In studies in mice, TMAO has been shown to cause atherosclerosis [39]. Cruciferous vegetables such as cabbage, broccoli, kale, and cauliflower are rich sources of fiber, lutein, flavonoids, phytosterols, folic acid, sulfur-containing glucosinolates, and vitamin C, each of which has been associated with reduced risk of various kinds of cancer [27]. Moreover, sulfur-containing glucosinolates can be converted to biologically active compounds such as indoles, nitriles, and isothiocyanates (ITCs) by β-thioglucosidases produced by the gut microbiome [61]. The glucosinolate of sulforaphane (SFN) is contained in several cruciferous vegetables but is present in highest concentration in broccoli [97]. ITCs can also exert epigenetic effects via modulation of DNA methylation. For example, DNA methyltransferases are downregulated by SFN, which can lead to site-specific CpG island demethylation in the telomerase reverse transcriptase (*TERT*) gene and a reduction of its cancer-sustaining expression [57].

### Cancer and the gut microbiome

#### Gastric cancer

Gastric cancers are often caused by infection with *Helicobacter pylori* which induces chronic gastritis and peptic ulcers [100]. Virulent strains harbor cytotoxin-associated gene pathogenicity island (CagPAI) that encode components of type IV secretion system (T4SS) [13]. Cag A oncoproteins are transported across the host cell membrane via the type IV secretion system T4SS. Studies have shown that *H. pylori* CagPAI dependently induces dephosphorylation of histone H3S10, H3 threonine 3, and deacetylation of H3K23 in gastric epithelial cells but does not affect nine other distinct histone modifications [14]. Microbial pathogens often manipulate host cell mechanisms through histone modifications. Hence, targeting these epigenetic modifications may be one of the important therapeutic strategies to stop the infection. Dietary phytochemicals with HDAC- or HAT-inhibiting properties may play a pivotal role in altering these histone modifications.

#### Colon cancer

Intestinal environmental changes are keys to progression toward adenoma and subsequently to colon cancer. A recent study has shown significant changes in fecal microbiota, especially decreases in fecal SCFA concentrations (acetate, propionate, and butyrate) and significant increases in fecal pH in colon cancer patients compared with healthy individuals [63]. It was also observed that the count of obligate anaerobes was significantly decreased in the case of colon cancer [56]. It has been hypothesized that it may be possible to prevent colorectal cancer by improving the intestinal environment, as the decrease in count of microbiota may be one of the causes of colon cancer development and progression. Adding probiotics to the diet is speculated as a way to reduce the risk of colon cancer [96]. In addition, adding cruciferous vegetables and green tea polyphenols may further bring about epigenetic modifications in the bacterial DNA or the genes that they target [34]. The microbiome may undergo significant changes in other forms of cancer such as breast cancer. It has often been observed that patients on chemotherapy have diarrhea and irritable bowel syndrome. Studies have shown considerable changes in the microbiome of the patients undergoing chemotherapy [84]. Therefore, improving the quality of the intestinal microbiota may be a therapeutic option to alleviate some of these side effects of chemotherapy and improve the quality of life of these patients. The alteration of the gut microbiome has been shown in several diseases including cancer as is summarized in Table 1.

**Table 1:** Alteration of gut microbiome in human diseases

Disease	Microbiome alteration	Reference
Irritable bowel syndrome	Increased ratio of the *Firmicutes* to *Bacteroidetes*	(Rajilić-Stojanović, Biagi et al. 2011) [70]
Crohn’s disease	Increased *Clostridium* species, *Ruminococcus torques*, and *E. coli*	(Martinez‐Medina, Aldeguer et al. 2006) [53]
Gastric cancer	*H. pylori* induces production of pro-inflammatory cytokines	(Tsuji, Kawai et al. 2003) [88]
Colorectal cancer	Abundance of *Fusobacteria* and *Coriobacteria*	(Castellarin, Warren et al. 2012) [9]
Obesity	Reduced ratio of *Bacteroidetes* to *Firmicutes*	(Ley, Bäckhed et al. 2005) [44]
Type 1 diabetes	Altered gut permeability to mannitol and lactulose	(Kuitunen, Saukkonen et al. 2002) [41]
Atherosclerosis	Metabolism of phospholipids by gut microbiota to trimethylamine-*N*-oxide	(Loscalzo 2011) [48]
Rheumatoid arthritis	Less *Bifidobacteria* and bacteria of the *Bacteroides-Porphyromonas-Prevotella*	(Vaahtovuo, Munukka et al. 2008) [90]
Autism	Higher number of *Clostridium* species known to produce tetanus neurotoxin (TeNT)	(Parracho, Bingham et al. 2005) [64]
		(Bolte 1998) [4]
Chronic fatigue syndrome	Lower levels of *Bifidobacteria* and small-intestinal bacterial overgrowth	(Logan, Venket Rao et al. 2003) [47]
Alzheimer’s disease	Excess ammonia production by gut microbiota	(Samsel and Seneff 2013) [76]

A study of the stool microbiome and metabolome differences between colorectal cancer (CRC) and healthy adults showed that several butyrate-producing bacteria were underrepresented in CRC samples, whereas mucin-degrading species *Akkermansia muciniphila* were about fourfold higher [95]. There is evidence to suggest that induction of pro-inflammatory responses by commensal bacteria contributes to tumor initiation and development [16]. Production of genotoxins and DNA-damaging superoxide radicals by *Enterococcus faecalis* are also mechanisms by which commensals can contribute to CRC development [23]. Irritable bowel syndrome including ulcerative colitis and Crohn’s disease also predisposes to CRC [72]. A study showed that infection with *Helicobacter hepaticus* in Rag 2^−/−^ mice led to increased inflammation and elevated TNF-α production [17]. SCFAs such as butyrate, acetate, and propionate, which are fermentation products from dietary fibers by colonic gut microbiota, can regulate neutrophil function and migration and exhibit anti-inflammatory activity [15]. Butyrate can induce TNF-α to potentiate cell death [35] and can, via miRNA-dependent *p21* gene expression activity, lead to colon cancer prevention [32].

#### Hormone-dependent, breast, ovarian, and endometrial cancer

There is an increasing interest in other cancers that may have directly or indirectly been influenced by the gut microbiome. The gut microbiome modulates estrogen metabolism and contributes to the proportion of re-circulated and excreted estrogen and estrogen metabolites [22]. The introduction of bacteria in germfree mice led to a significant rise in the reproductive capacity of the germfree mice [81]. The capacity of reproduction of germfree mice is generally lower. However, addition of bacteria showed normalization in the estrous cycle and a significant rise in copulation and implantation rates. Also, there were studies that showed that a reduction in the population of specific gut bacteria in humans causes increased fecal excretion of conjugated estrogens and decreases in urinary estrogens. Human fecal extracts metabolize plasma estrone (E1), 17β-estradiol (E2), and 16α-hydroxyestrone in vitro and lead to the inter-conversion of E1 and E2 and the reduction of 16α-hydroxyestrone to estriol (E3), 16-oxoestradiol to 16-epiestriol, and 15α-hydroxyestrone 15α-hydroxyestradiol. The human gut microbiome can influence the estrabolome. This is due to the presence of aggregate of enteric bacterial genes whose products are capable of metabolizing estrogen. Some bacterial species possess β-glucuronidases and β-glucuronides which are the enzymes involved in estrogen deconjugation and conjugation [68]. Deconjugation reactions may result in greater reabsorption of free estrogens, leading to the development of estrogen-driven cancers such as breast, ovarian, and endometrial cancers. Phase II hepatic conjugation reactions of E1 and E2 include methylation via catechol-*O*-methyltransferase (COMT) and glucuronidation via uridine 5′-diphospho-glucuronosyltransferase. COMT converts genotoxic catechol estrogens to their inactive methoxy derivatives, 2-MeOE2 and 4-MeOE2. Thus, consumption of certain plant-based diet (such as tomato) containing high levels of *O*-methyltransferases may reduce exposure to potentially mutagenic estrogen metabolites [50]. Daidzein is converted by gut microflora into dihydrodaidzein, S-(−)equol (70 %) and *O*-desmethylangolensin (5–20 %) [37]. Studies on soy isoflavones have shown that the ability to produce equol varies (only 30–50 % of US populations are equol producers) and is primarily dependent on intestinal bacterial composition of an individual [3, 43]. It has been shown that the amount of urinary equol excretion is correlated with a reduced risk of breast cancer, and epigenetic modifications may play a role in impacting cancer secondary to changes in metabolites [74]. The study of these interactions may provide new avenues for therapeutic research. Colonization with specific bacteria that can modulate levels of certain metabolites may be another way to target diseases such as cancer.

#### Liver cancer

Toll-like receptors (TLR4) are activated by lipopolysaccharide (LPS) from gut bacteria and may contribute to injury and inflammation-driven tumor promotion in the liver [11]. Epigenetic regulation of *TLR4* gene expression in intestinal epithelial cells can act as one mechanism for maintaining intestinal homeostasis by suppressing excessive responses to the commensals and regulating mucosal inflammation in the gut [87]. Dietary regulation of epigenetic modification may be useful for the prevention of liver cancer. SCFAs produced by gut microbiota bind G protein-coupled receptor 43 (GPCR43), and this interaction affects inflammatory responses [46]. GPR43-deficient (*Gpr43*^*−/−*^) mice showed exacerbated inflammation in models of colitis, arthritis, and asthma [92]. These effects appear to relate to increased production of inflammatory mediators by *Gpr43*^*−/−*^ immune cells and increased immune cell recruitment [93].

#### Lung cancer

Chronic inflammation plays a central role in the pathogenesis of chronic obstructive pulmonary disease (COPD) and lung tumorigenesis. Chromatin modifications and other epigenetic changes, angiogenesis, and apoptosis are crucial in the development of COPD and lung cancer [98]. Recent studies have established a role of miRNA as a pathogenic link between COPD and lung cancer [82]. Hence, emerging science should explore the relationship between the gut microbiome, its various metabolites, and cancer epigenetics (Table 2).

**Table 2:** Gut microbiota and its emerging science in cancer epigenetics

Cancer	Link to gut microbiome	Link to epigenetics	Possible therapy	Reference
Gastric	*H. pylori* causes gastric pathology by injecting about 128 kDa Cytotoxin-associated gene A (Cag A) protein which enhanced c-Myc and DNMT3B expression	*H. pylori* increased histone H4 acetylation in the promoter region of *p21* thereby increasing *p21* expression	Dietary compounds that have HAT-inhibiting activities	(Hayashi, Tsujii et al. 2012) [26]
				(Ding, Goldberg et al. 2010) [13]
				(Fehri, Rechner et al. 2009) [21]
Colon	Elevated TNF-α expression in the colon	TNF-α-suppressed differentiation and potentiated cell death induced by butyrate (NaBt) in both adenocarcinoma HT-29 and fetal FHC human colon cells in vitro	Sodium butyrate which is an HDAC inhibitor can induce TNF-α to potentiate cell death	(Hýžd’alová, Hofmanova et al. r2008) [35]
				(Erdman, Rao et al. 2009) [17]
Estrogen-dependent cancers	Bacterial species possess β-glucuronidases and β-glucuronides that participate in estrogen conjugation and deconjugation	Phase II hepatic conjugation reactions of E1 and E2 include methylation via catechol-*O*-methyltransferase, glucuronidation via uridine 5′-diphospho-glucuronosyltransferase	Certain plant-based dietary compounds contain *O*-methyltransferase enzymes that can inactivate catechol estrogens	(Plottel and Blaser 2011) [68]
				(Mageroy, Tieman et al. 2012) [50]
Liver	TLR4 activation by LPS from gut bacteria contributes to tumor promotion	Epigenetic regulation of TLR4 gene expression in intestinal epithelial cells (IECs) can act as one mechanism for maintaining intestinal homeostasis by suppressing excessive responses to the commensals and regulating mucosal inflammation in the gut		(Dapito, Mencin et al. 2012) [11]
				(Takahashi, Sugi et al. 2011) [87]
Lung	SCFAs produced by gut microbiota bind GPCR43 which affects inflammatory responses. GPR43-deficient (*Gpr43* ^*−/−*^) mice showed exacerbated or unresolving inflammation in models of colitis, arthritis, and asthma	SCFAs are known to have HDAC-inhibiting activities	SCFAs are produced by fermentation of carbohydrates by gut microbiota	(Maslowski, Vieira et al. 2009) [54]

## Conclusion

The indigenous microbiota plays a very important role in either directly or indirectly affecting epigenetic processes in the body. The LMW molecules produced by the microbiota of the gut participate in various molecular pathways and thus are implicated in the etiology of many diseases. Diet also plays a vital role in maintaining the health of an individual as the dietary components directly interact with the LMW molecules produced by the microbiota. Thus, studying the inter-relationship between the human gut microbiota, their genetic makeup, the epigenome, and dietary components may offer new therapeutic options for treating diseases. Studies on family clusters of nonagenarian and centenarian siblings have shown that apart from inheriting exceptional gene groups, behavior and lifestyle have contributed to their aging well phenotype [71]. Thus, the study of this interrelation is important for disease prevention. The Human Microbiome Project aims at a better understanding of the roles of microbes on human biology including their relationship with health and disease [91]. The human intestinal microbiome will pave the way leading to a new frontier in human biology, in which the human genome and the bioactive products from the intestinal microbiome are tightly linked together as an integral part of the “human metagenome.” Bioactive products such as LMW compounds may bring about epigenetic changes that directly and/or indirectly modify various epigenetic pathways through hormonal intermediates or immunological messengers. The study of epigenetic changes dependent on the microbiome is especially important for the study of cancer. A good animal model is essential for the study of the interactions among diet, gut microbiome, and cancer. The majority of anaerobic bacteria present in our digestive tract cannot be cultivated on existing media. Zebrafish can be a good model for microbiome studies as they share a great deal of homology with mammals. Most zebrafish genes have mammalian orthologs, and most epigenetic regulators are highly conserved: there are 75 and 92 % identity between zebrafish and human *DNMT1* and *HDAC1*, respectively [60]. Some other useful models for exploring host-microbiota interactions are fruit fly and yeast. However, most extensively used models for the study of cancer are rodents. Human fecal samples can be transplanted into rodents that are raised in a germfree environment where they have no exposure to microbes. Such animals, called *gnotobiotic mice,* are helpful in studying microbial community compositions by varying diet and disease parameters. These mice can harbor the microbial community in humans and hence are called “humanized mice.” It is, however, important to note that although germfree mice do not harbor live bacteria, they still are exposed to a minimum amount of microbes in food, water, and bedding. They can be further induced with cancer cell line xenografts, and different dietary interventions can be introduced for effective study of intestinal microbial epigenetics [10]. Epigenetic dietary phytochemicals such as SFN from broccoli sprouts, EGCG from green tea, or genistein from soy products may positively enhance the interaction among metabolism, diet, gut microbiome, and epigenome. This can be very useful to treat a variety of cancers such as colon, lung, or even breast cancers where the current chemotherapy drugs such as doxorubicin/docetaxel can strip the body of its essential microbiome. Administering an “epigenetic diet” along with conventional treatment can be effective in enhancing the quality of the microbiome in cancer patients. Thus, by manipulating both the microbiome and the diet, we may discover novel therapies that act through epigenetic pathways and add to our armamentarium against cancer.

## Abbreviations

ASD: autism spectrum disorder
CagPAI: cytotoxin-associated gene pathogenicity island
COPD: chronic obstructive pulmonary disease
CRC: colorectal cancer
CREB: cAMP response element-binding protein
DNMT: DNA methyltransferase
EGCG: epigallocatechin
HAT: histone acetyltransferase
HDAC: histone deacetylase
HMP: Human Microbiome Project
HMT: histone methyltransferase
ITC: isothiocyanate
LMW: low molecular weight
MBD: methyl binding domain
MECP2: methyl CpG binding protein 2
piRNA: PIWI-interacting RNA
PPA: propionic acid
SCFA: short-chain fatty acid
SFN: sulforaphane
TLR: toll-like receptor
TMAO: trimethylamine-*N*-oxide
α-KG: alpha ketoglutarate
